# Evaluating the relationship between conditional cash transfer programme on preterm births: a retrospective longitudinal study using the 100 million Brazilian cohort

**DOI:** 10.1186/s12889-024-18152-2

**Published:** 2024-03-05

**Authors:** Naiá Ortelan, Márcia Furquim de Almeida, Elzo Pereira Pinto Júnior, Nivea Bispo, Rosemeire L. Fiaccone, Ila Rocha Falcão, Aline dos Santos Rocha, Dandara Ramos, Enny S. Paixão, Rita de Cássia Ribeiro-Silva, Laura C. Rodrigues, Mauricio L. Barreto, Maria Yury T. Ichihara

**Affiliations:** 1 Center for Data and Knowledge Integration for Health (CIDACS), Gonçalo Moniz Institute (IGM), Oswaldo Cruz Foundation (FIOCRUZ-BA), Salvador, Bahia Brazil; 2 School of Public Health, University of São Paulo (USP), Sao Paulo, Brazil; 3 Institute of Mathematics and Statistics, Federal University of Bahia (UFBA), Salvador, Bahia Brazil; 4 Collective Health Institute, Federal University of Bahia (UFBA), Salvador, Bahia Brazil; 5 Faculty of Epidemiology and Population Health, London School of Hygiene and Tropical Medicine, London, UK; 6 School of Nutrition, Federal University of Bahia (UFBA), Salvador, Bahia Brazil; 7 Center for Data and Knowledge Integration for Health (CIDACS), Oswaldo Cruz Foundation. Edifício Tecnocentro, Rua Mundo, 121, Trobogy, Salvador, Bahia 41745-715 Brazil

**Keywords:** Preterm birth, Conditional cash transfer, Bolsa Familia Programme, Income redistribution, 100 million Brazilian cohort, Prenatal care, Propensity score

## Abstract

**Background:**

Preterm births increase mortality and morbidity during childhood and later life, which is closely associated with poverty and the quality of prenatal care. Therefore, income redistribution and poverty reduction initiatives may be valuable in preventing this outcome. We assessed whether receipt of the Brazilian conditional cash transfer programme - Bolsa Familia Programme, the largest in the world - reduces the occurrence of preterm births, including their severity categories, and explored how this association differs according to prenatal care and the quality of Bolsa Familia Programme management.

**Methods:**

A retrospective cohort study was performed involving the first live singleton births to mothersenrolled in the 100 Million Brazilian Cohort from 2004 to 2015, who had at least one child before cohort enrollment. Only the first birth during the cohort period was included, but born from 2012 onward. A deterministic linkage with the Bolsa Familia Programme payroll dataset and a similarity linkage with the Brazilian Live Birth Information System were performed. The exposed group consisted of newborns to mothers who received Bolsa Familia from conception to delivery. Our outcomes were infants born with a gestational age < 37 weeks: (i) all preterm births, (ii) moderate-to-late (32–36), (iii) severe (28–31), and (iv) extreme (< 28) preterm births compared to at-term newborns. We combined propensity score-based methods and weighted logistic regressions to compare newborns to mothers who did and did not receive Bolsa Familia, controlling for socioeconomic conditions. We also estimated these effects separately, according to the adequacy of prenatal care and the index of quality of Bolsa Familia Programme management.

**Results:**

1,031,053 infants were analyzed; 65.9% of the mothers were beneficiaries. Bolsa Familia Programme was not associated with all sets of preterm births, moderate-to-late, and severe preterm births, but was associated with a reduction in extreme preterm births (weighted OR: 0.69; 95%CI: 0.63–0.76). This reduction can also be observed among mothers receiving adequate prenatal care (weighted OR: 0.66; 95%CI: 0.59–0.74) and living in better Bolsa Familia management municipalities (weighted OR: 0.56; 95%CI: 0.43–0.74).

**Conclusions:**

An income transfer programme for pregnant women of low-socioeconomic status, conditional to attending prenatal care appointments, has been associated with a reduction in extremely preterm births. These programmes could be essential in achieving Sustainable Development Goals.

**Supplementary Information:**

The online version contains supplementary material available at 10.1186/s12889-024-18152-2.

## Background

Preterm birth (PTB) - live birth occurring before 37 completed weeks of gestation [1] - is associated with increased mortality and morbidity not only in the immediate neonatal period but also throughout infancy, childhood, and even adulthood, resulting in heightened costs to health systems [2–6]. PTB can be further categorized into moderate-to-late (32 to < 37 weeks), severe (28 to < 32 weeks), and extreme (< 28 weeks) [1]. These subdivisions are important since the reduction in gestational age is associated with the survival of the newborn and neonatal complications [4, 7]. 

The most recent analysis showed that the worldwide PTB rate rose from 9.8% in 2000 to 10.6% in 2014, equating to 14.8 million liveborn preterm babies. Most PTBs occur in low- and middle-income countries (LMICs) [1, 3, 8]. In Brazil, between 2012 and 2019 the proportion of overall preterm births decreased, ranging from 10.87 to 9.95%, with the lowest proportion in 2015 (9.77%). The proportion of extreme, severe, and moderate-to-late PTB were, respectively, 0.09%, 0.53%, and 9.49% [9]. 

An essential strategy for mitigating the risk of PTB is ensuring adequate prenatal care [10, 11]. Several studies employing prenatal care utilization indices have identified an association between inadequate prenatal care and adverse birth outcomes, particularly PTB [10–16]. However, when exploring trends in prenatal care and birth outcomes, diverse methods have been used to assess the adequacy of prenatal care [17, 18], yielding null [19, 20] or opposite findings as a greater risk of PTB among mothers with a high number of prenatal care visits (the “adequate-plus” category) [21]. This result can be explained by a bias in the definition of this index, since higher-risk pregnancies, in which the number of observed visits is greater than expected, are assigned to a “better prenatal care” level [21, 22]. 

Furthermore, evidence shows that PTBs are associated to poverty-related risk factors [8, 23]. Compared to pregnant women with higher incomes, those living in poverty experience elevated levels of stress [24, 25], inadequate nutritional intake [24, 26], higher levels of smoking during pregnancy [24, 25, 27], poorer maternal health [24], and poorer access to adequate prenatal care [24]. In low-income settings, nearly half of the infants born at or below 32 weeks (2 months early) die due to a lack of feasible, cost-effective care, such as warmth, breastfeeding support, and basic interventions for infections and respiratory issues. Shockingly, over 90% of extremely preterm babies born in low-income countries die within the first few days of life, while less than 10% face a similar fate in high-income settings. These statistics underscore the profound impact of socio-economic factors on the survival rates of preterm infants [28].

Therefore, income redistribution initiatives might reduce PTBs cases and programs providing income supplements to low-income pregnant women are an increasingly common initiative, especially in LMICs, as a strategy for social protection and poverty reduction [29]. There are unconditional cash transfer programs (UCTs), which provide cash directly to eligible households based on specific inclusion criteria, and conditional cash transfer programs (CCTs), which require the fulfilment of specific education and health-related conditions for continued receipt [29, 30]. Other CCTs, such as India’s Janani Suraksha Yojana (JSY) and Nepal’s Safe Delivery Incentive Program, transfer cash only for use of specific services, such as health facility–based delivery [31]. Well-studied CCTs in LMICs, including the Bolsa Familia (BF) Programme in Brazil, acknowledged as the world’s largest CCT in terms of coverage and financing, Progresa and Opportunidades programs in Mexico, and the JSY in India, are associated with improved birth outcomes such as increased birthweight [32, 33], better outcomes in child health, growth, and development [34], and decreased neonatal/infant mortality [35–37]. Ramos et al. (2021) [35] also observed a more pronounced reduction in child mortality (aged 1–4 years) among children born preterm in Brazil who were beneficiaries of the BF. Moreover, increased income from the Earned Income Tax Credit (EITC) in the United States (US) has been associated with more favorable birth outcomes [38, 39]. 

However, evidence on the effect of CCTs on PTB is lacking, especially in LMICs and when considering PTB subgroups. In Canada [26, 40, 41] and US [42], unconditional prenatal benefits provided to low-income pregnant women were associated with a lower risk of PTB. A recent Brazilian study [43] showed that children born to mothers receiving BF were less likely to be born before 37 or 28 weeks of gestation. In Uruguay [44] and another study in the US [45], it was observed that social assistance programs had no observable effect on PTBs. In the present study, we hypothesized that BF assistance could reduce the likelihood of PTB. Therefore, we investigated the association between receiving BF assistance throughout pregnancy and the occurrence of PTB, considering both overall and their severity levels (moderate-to-late, severe, and extreme). Additionally, we aimed to explore whether the observed association varies based on prenatal care and the quality of BF management in the mothers’ municipalities. It is crucial to note that, although mothers were not primiparous upon enrollment into the cohort, we only considered the first child born after their registration. This approach was implemented to prevent biasing our results.

## Methods

All of the methods and analyses were described in the previously published research protocol [46]. 

### Study design and participants

In this retrospective cohort study, we included the first live singleton births to mothers aged between 10 and 49 years enrolled in the 100 Million (100 M) Brazilian Cohort [47] from 2004 to 2015, who had at least one child before cohort enrollment. Only the first child born after registration, but born from 2012 onward, was included. We can affirm that between 2004 and 2015, these multiparous mothers did not undergo additional pregnancies since we obtained the birth order of their children from the original dataset, retaining only the first child born during the cohort period. Moreover, our study used sociodemographic information from 2004, the year when the BF was implemented; and live birth information from 2011, due to a modification in birth certificates. This modification included essential variables, such as gestational age recorded as a continuous variable in completed weeks (previously reported in broad intervals of gestational weeks, hindering consistent classification and leading to an underestimation of prematurity rates); the mother’s date of birth, thereby improving the linkage process; and the number of prenatal consultations as a quantitative variable.

We excluded: (i) births between 2004 and 2011, given the impossibility of categorizing the outcome by gestational age in completed weeks; (ii) births from primiparous women due to their increased risk of PTB compared to women with a previous term birth [48, 49] and those that were not the first child following the mother’s enrollment (Table S1 in Additional file 1); (iii) inconsistent data (birthdates, birth weights for gestational age [50], inter-birth intervals under 196 days) and unmeasured outcomes; and (iv) non-viable newborns (born before 22, weighing less than 500 g or over than 6,999 g) [51, 52] or presenting conditions associated with PTB (congenital abnormal births [53] and multiple pregnancies [54]).

Further details on the definition of the study sample are provided in Additional file 1.

This cohort study was approved by the research ethics committee of the Federal University of Bahia, Salvador, Brazil, and followed Strengthening the Reporting of Observational Studies in Epidemiology (STROBE) reporting guideline. As no personally identifiable information was included in the dataset used for analysis, the need for informed consent was waived in accordance with the National Commission of Ethics in Research (CONEP).

### Data sources and linkage

The 100 M cohort is a population-based cohort compiled from the Unified Registry for Social Programs (CadÚnico), which holds data on individuals at their first registration on this Brazilian national social program registry. CadÚnico is a unique registry for selecting low-income families requesting support from over 20 social protection programs in Brazil. Once a family applies, it becomes eligible to receive one or more benefits (with a maximum of 5), depending on their income threshold and composition. Therefore, our database reflects the poorest half of the Brazilian population. The cohort database is comprised of records containing the socioeconomic data of 114,008,179 low-income individuals who have applied for social assistance programs through CadÚnico, representing approximately 55% of the entire Brazilian population [47, 55]. 

**Fig. 1:**
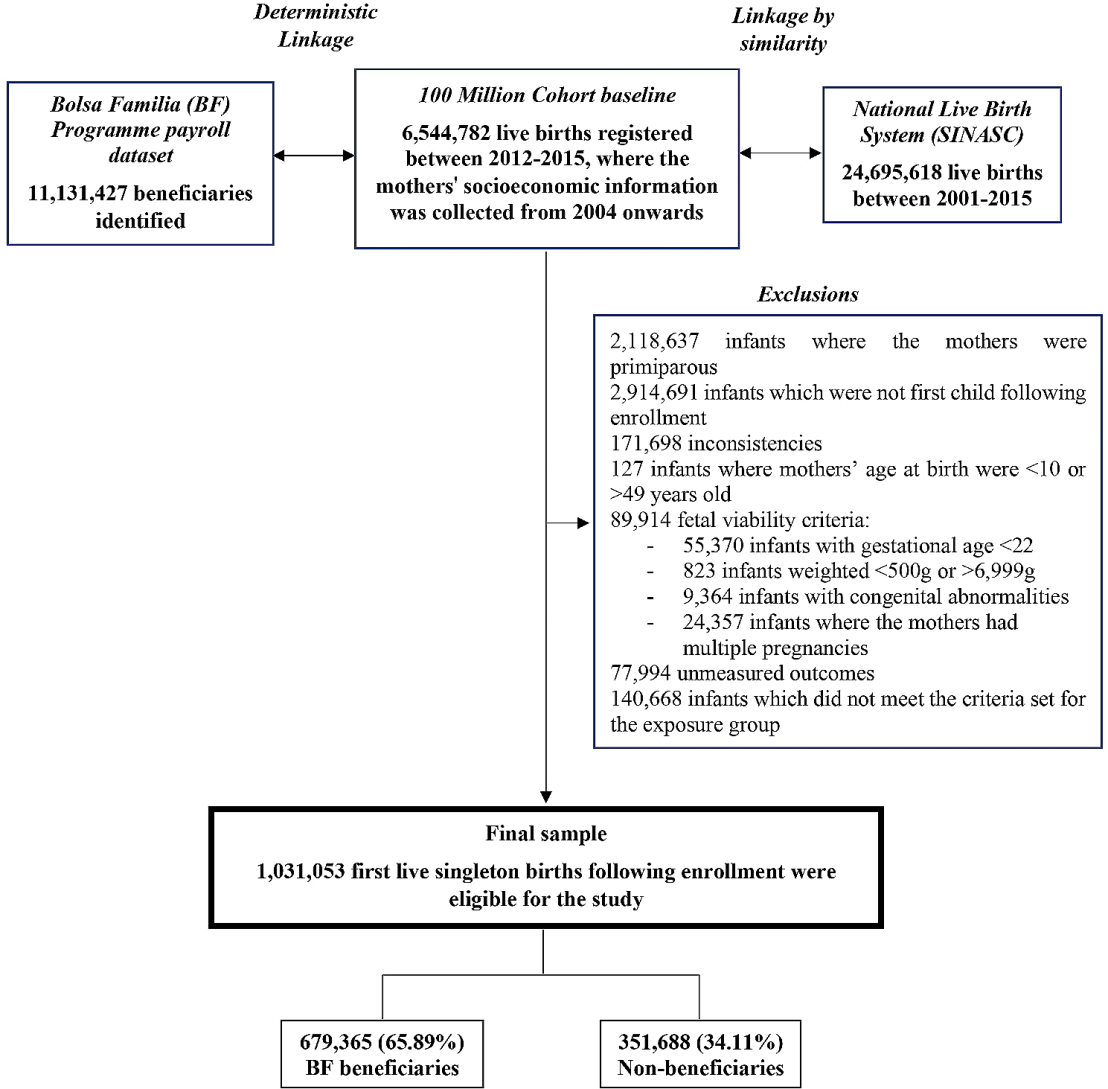
Flowchart of the study population selection

The 100 M cohort baseline was linked to two other databases (Fig. 1): the BF Programme payroll database (2004–2015), to identify the beneficiaries during pregnancy; and the Brazilian Live Birth Information System (SINASC, 2012–2015), to assess relevant maternal and gestational data. Finally, the merge with the database of the municipal Decentralized Management Index (DMI) of the Bolsa Familia Program and the CadÚnico was performed to obtain aggregated data at the municipal level.

Deterministic, exact linkage was performed to link the cohort baseline and the BF payroll datasets, using a unique identifier called a social identification number (NIS). To link the cohort baseline and the SINASC data we used the CIDACS record linkage algorithm (CIDACS-RL), an innovative linkage tool based on a combination of indexing search and scoring algorithms, with high accuracy developed by Center for Data and Knowledge Integration for Health (CIDACS) [56]. The following variables were used for this linkage: mother’s name, maternal age or date of birth, and mother’s municipality of residence. Missing, implausible names, and duplicates, were excluded. The linkage between the cohort baseline and SINASC is detailed elsewhere [57, 58]. 

From 2004 to 2015, 20,232,461 (57.3%) live births were linked, with a high estimated accuracy, which increased after 2011 (Table S2 in Additional file 1) [32, 58]. The data were stored on secure servers at the CIDACS, where all linking procedures were performed in compliance with all ethical and legal standards [57, 59]. 

### BF programme characteristics

For a family to be a BF beneficiary, it must be registered on CadÚnico. The BF eligibility criteria are CadÚnico registered families’ per capita income and composition (such as the presence of children adolescents, and pregnant women).

Considering the income threshold for the study period, families with a monthly per capita income of up to BRL 77 (income cut-off point for 2014/2015, equivalent to USD 15.74) were considered extremely poor and eligible, independent of their composition [46]. Similarly, families with a monthly income equal to or below three minimum wages (BRL2,364.00, equivalent to USD483.32 in 2015) were considered eligible. Poor families were eligible to receive BF assistance if their per capita income was between BRL 77.01 and BRL 154.00 (income cut-off point for 2014/2015, equivalent to USD31.49) and have at least 1 individual from a priority group, such as children or teenager aged under 17 years, or a pregnant or lactating woman in the household [60]. The estimate of low-income families in each municipality is calculated based on National Household Sample Survey (PNAD) [61] data, which is used as a guide to implement BF, but is not a ceiling, or threshold, for expenditure.

Preferably, BF cash payments are transferred to women. Once a family becomes a BF beneficiary, it must comply with specific program requirements (conditionalities) to remain on the programme. These criteria include the imperative of consistent school attendance and use of health care services throughout childhood (including maintaining an up-to-date vaccination schedule), during pregnancy (attending prenatal consultations), and in the postpartum period [62]. Further details on BF characteristics related to variable benefits provided to breastfeeding mothers and pregnant women are described in the Additional file 1.

### Variables of interest

**Fig. 2:**
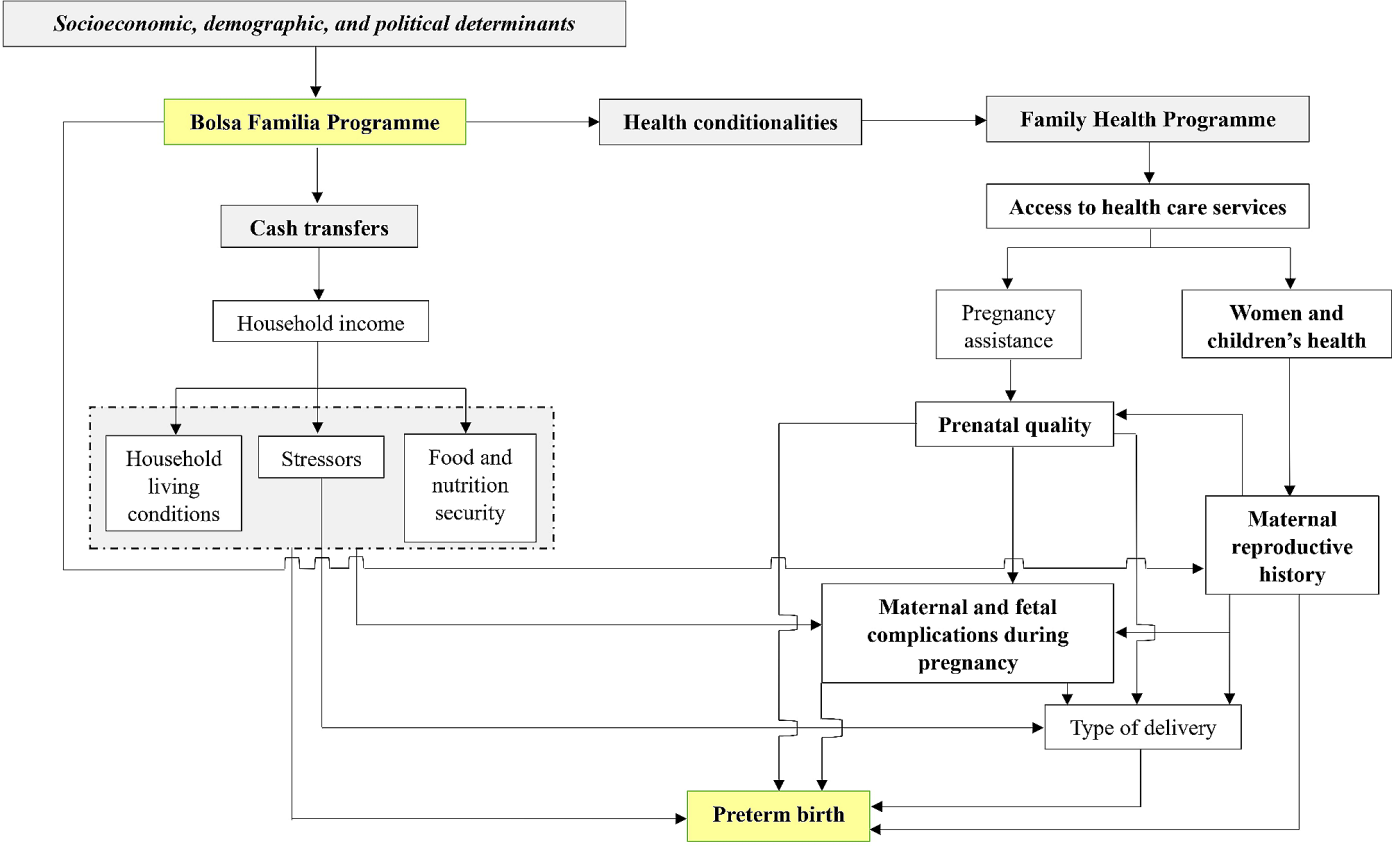
Theoretical model of the potential pathways by which the Bolsa Familia (BF) Programme may affect preterm births

The variables of interest (Table 1) below are part of the proposed framework in Fig. 2.

**Table 1:** Main components of the 100 Million Brazilian Cohort, sources of data, and relevant variables

Component	Data source	Follow-up period		Variable
		Original Cohort	Cohort used	
Cohort baseline	Unified Registry for Social Programs (CadÚnico)	2001–2015	2004^a^-2015	Cohort time^c^ (calculated by the difference between the child’s date of birth and the mother’s date of enrollment): ≥5; <5 years
				Self-reported maternal race/skin color ^c^: white; black + mixed-race/brown; and indigenous
				Geographical region^c^: South; North; Northeast; Southeast; and Central-West
				Household location^c^: urban; rural
				Household conditions^c, d^: all favorable conditions; 1 unfavorable condition; 2 unfavorable conditions; 3 unfavorable conditions; 4–5 unfavorable conditions
				Overcrowding^c^ (calculated by dividing the number of individuals living in the house and the number of rooms): ≤2; >2 people per room
Exposure	Bolsa Familia (BF) Programme payroll database	2004–2015	2004–2015	Beneficiary group (exposed): non-beneficiary; BF beneficiary
Outcomes, maternal and gestational data	Brazilian Live Birth Information System (SINASC)	2001–2015	2012^b^-2015	Gestational age, in weeks (used to classify the following four outcomes): 1. Preterm birth (< 37 weeks) versus (vs.) at-term newborns (37–42 weeks); 2. Moderate-to-late preterm birth (32–36 weeks) vs. at-term newborns (37–42 weeks); 3. Severe preterm birth (28–31 weeks) vs. at-term newborns (37–42 weeks); 4. Extreme preterm birth (< 28 weeks) at-term newborns (37–42 weeks)
				Maternal education^c^: 0–7; 8–11; ≥12 years
				Marital status^c^: marriage/civil partnership; single/divorced/widow
				Maternal age^e^: 20–34; 10–19; 35–49 years
				Number of prenatal visits - used to define adequacy of prenatal care: adequate; inadequate
				Type of delivery^e^: vaginal birth; c-section
Aggregate data at municipal level	Municipal Decentralized Management (DMI) index	2006–2015	2012–2015	Tertile of DMI: 1st tertile (worst); 2nd tertile; 3rd tertile (best)

^a^ From the year in which the Bolsa Familia Programme was implemented

^b^ From the year when the gestational age has been recorded in completed weeks and no longer in aggregate form

^c^ Baseline characteristics of the child’s mother and living conditions as regressors in the Propensity Score model

^d^ Created from five binary variables referring to household conditions, considering the reference category as favorable conditions, as follows - household building materials (brick/rubble; others); sewage (city public system; others); water supply (public network; others); garbage disposal (city collection; no collection-burned/buried); and electrical energy (meter for private or community use; no meter)

^e^ Variable used for adjustment

The exposed group comprised live births to mothers who continuously received BF throughout the entire pregnancy period, from conception to delivery. Unexposed group included live births to mothers who did not receive BF benefit at any time, or until childbirth. Live births to mothers who discontinued receiving the benefit were not considered in the analysis.

There are four outcomes, where we compared at-term newborns (37–42 gestational weeks – reference category) to (i) overall, < 37 weeks; (ii) moderate-to-late, 32 to < 37 weeks; (iii) severe, 28 to < 32 weeks; and (iv) extreme, < 28 weeks, PTBs.

Sociodemographic and household information at the individual level (collected before receipt of BF) were taken from the cohort baseline and the characteristics of the mother and the newborn from SINASC records (Table 1).

We used two variables in the subgroup analysis. First, adequacy of prenatal care following the Brazilian Ministry of Health Prenatal Care and Birth Humanization Programme (PHPN), which recommends three, four, five, and six prenatal visits for babies born between 22 and 27 weeks, 28 and 33 weeks, 34 and 37 weeks, and over 37 weeks, respectively [63]. Those who complied with the recommendation were classified as adequate (category 1), and those who did not as inadequate (category 0).

Second, we considered the municipal Decentralized Management Index, an administrative indicator of the quality of BF and CadÚnico management at the municipal level, related to updating records, success in attaining families in extreme poverty, and following-up health, education, and social control conditionalities by programme beneficiaries [61]. It varies from 0 (worst) to 1 (best) and the municipalities receive financial support from the federal government via payment of an amount per registered family, weighted by the quality indicator [64]. We classified municipalities according to DMI tertile.

### Statistical analysis

Since eligibility to receive BF is determined by per capita income and a set of family socioeconomic characteristics, rigorous control in the analysis is required. We followed a kernel matching approach to choose a set of BF non-beneficiary mothers inside the cohort, and this allowed us to balance the two groups on observable characteristics.

Our analysis combines propensity score (PS) kernel matching and weighted logistic regressions. First, we used a logit model to estimate the probability of receiving BF assistance during pregnancy, mostly based on the mother’s baseline sociodemographic characteristics. Given that estimated propensity scores are used to adjust for measured confounding [65], we estimated the PS of receiving BF assistance during pregnancy, considering the sociodemographic and household covariates in the PS model (note “c” of Table 1). Having estimated the PS, we performed a kernel matching procedure, which establishes a non-parametric approach, using the weighted averages of all individuals in the control group, to construct the counterfactual outcome. Additional weight is given to units close to the one that needs to be matched [66]. 

In the final logistic models at the first stage of the kernel weighted, we adjusted for relevant maternal (age) and perinatal (type of delivery) conditions.

The subgroup analysis was established by our research protocol [46]. We investigated whether the effect of receiving BF during pregnancy and a preterm birth varied according to the adequacy of the mother’s prenatal care and the municipal decentralized management index. Kernel-weighted logistic models were calculated separately within each adequacy of the prenatal care subgroup, to conduct the first subgroup analysis. For the second subgroup, we ranked municipalities into DMI tertiles and all the analysis steps separately, including the PS estimate, kernel matching, and final weighted logistic models, providing separate estimates.

To formally assess whether the association between receiving BF benefit and PTB outcomes varies across subgroups, we conducted a statistical test for interaction by including BF status × subgroup indicator terms in our subgroup models (see Interaction test in Additional file 1). The analysis of heterogeneity in the BF association with PTB across subgroups relied on the statistical significance (*p* *<* 0.05) of the interaction terms [67], and we applied the likelihood ratio test to evaluate the difference between nested models. It is important to note that the model without the interaction term is nested within the model with the interaction term. The use of interaction terms results in numerous hypothesis tests, especially in the context of categorical covariates. To account for that, we applied the Bonferroni correction to adjust *p*-values for multiple comparisons within factor variable terms. We checked robustness by using the inverse probability of treatment weighting (IPTW) as an alternative approach, with the association between BFP participation and PTB outcomes estimated through logistic models with weights equal to the PS/(1-PS) for non-beneficiaries, and equal to 1 for the beneficiaries, with further adjustment for maternal and perinatal conditions.

A case-control study was conducted using the 100 M cohort to check the robustness of these findings, considering that severe and extreme PTBs are rare events with a 0.94% and 0.38% prevalence, respectively (Table 2). All the analyses were performed using Stata version 15.0.

## Results

Of the 1,031,053 first live births eligible for the study, 679,365 (65.9%) of the mothers received BF throughout pregnancy, and 351,688 (34.1%) did not (Fig. 1). The prevalence of overall and moderate-to-late PTBs was slightly higher among beneficiaries, compared to non-beneficiaries – except for those born moderate-to-late whose mothers had inadequate prenatal care - while severe and extreme PTBs were lower among beneficiaries compared to non-beneficiaries – except for those severe PTB whose mothers had adequate prenatal care and lived in 2nd tertile of DMI (Table 2).

**Table 2:** Description of preterm birth (PTB) outcomes among Bolsa Familia (BF) beneficiaries and non-beneficiaries in Brazil (2012–2015), by the subgroups of prenatal care adequacy and municipal BF programme Decentralized Management Index (DMI).

Subgroup	N (%)	Outcome							
		Preterm birth (PTB)		Moderate-to-late PTB		Severe PTB		Extreme PTB	
		No (37–42 weeks)	Yes (22–36 weeks)	No (37–42 weeks)	Yes (32–36 weeks)	No (37–42 weeks)	Yes (28–31 weeks)	No (37–42 weeks)	Yes (< 28 weeks)
Brazil	BF	602,595 (88.7)	76,770 (**11.3**)	602,595 (89.7)	69,082 (**10.3**)	602,595 (99.07)	5,633 (**0.93**)	602,595 (99.66)	2,055 (**0.34**)
	Non-BF	313,810 (89,2)	37,878 (**10.8**)	313,810 (90.4)	33,378 (**9.6**)	313,810 (99.04)	3,043 (**0.96**)	313,810 (99.54)	1,457 (**0.46**)
	Total	916,405 (88.9)	114,648 (11.1)	916,405 (89.9)	102,460 (10.1)	916,405 (99.06)	8,676 (0.94)	916,405 (99.62)	3,512 (0.38)
Adequate prenatal care	BF	5,894 (75.3)	1,936 (**24.7**)	5,894 (80.1)	1,467 (**19.9**)	5,894 (95.42)	283 (**4.58**)	5,894 (96.94)	186 (**3.06**)
	Non-BF	2,094 (77.4)	612 (**22.6**)	2,094 (83.0)	429 (**17.0**)	2,094 (95.84)	91 (**4.16**)	2,094 (95.79)	92 (**4.21**)
	Total	7,988 (75.8)	2,548 (24.2)	7,988 (80.8)	1,896 (19.2)	7,988 (95.53)	374 (4.47)	7,988 (96.64)	278 (3.36)
Inadequate prenatal care	BF	2,200 (79.4)	569 (**20.6**)	2,200 (84.2)	412 (**15.8**)	2,200 (96.92)	70 (**3.08**)	2,200 (96.20)	87 (**3.80**)
	Non-BF	899 (75.0)	300 (**25.0**)	899 (81.9)	198 (**18.1**)	899 (95.33)	44 (**4.67**)	899 (93.94)	58 (**6.06**)
	Total	3,099 (78.1)	869 (21.9)	3,099 (83.6)	610 (16.4)	3,099 (96.45)	114 (3.55)	3,099 (95.53)	145 (4.47)
Worst DMI − 1st tertile	BF	238,525 (88.9)	29,935 (**11.1**)	238,525 (89.9)	26,724 (**10.1**)	238,525 (99.03)	2,329 (**0.97**)	238,525 (99.63)	882 (**0.37**)
	Non-BF	151,733 (89.2)	18,449 (**10.8**)	151,733 (90.4)	16,194 (**9.6**)	151,733 (98.99)	1,542 (**1.01**)	151,733 (99.53)	713 (**0.47**)
	Total	390,258 (89.0)	48,384 (11.0)	390,258 (90.1)	42,918 (9.9)	390,258 (99.02)	3,871 (0.98)	390,258 (99.59)	1,595 (0.41)
2nd tertile	BF	215,978 (88.7)	27,532 (**11.3**)	215,978 (89.7)	24,780 (**10.3**)	215,978 (99.08)	2,015 (**0.92**)	215,978 (99.66)	737 (**0.34**)
	Non-BF	116,392 (89.4)	13,792 (**10.6**)	116,392 (90.5)	12,185 (**9.5**)	116,392 (99.11)	1,049 (**0.89**)	116,392 (99.52)	558 (**0.48**)
	Total	332,370 (88.9)	41,324 (11.1)	332,370 (90.0)	36,965 (10.0)	332,370 (99.09)	3,064 (0.91)	332,370 (99.61)	1,295 (0.39)
Best DMI – 3rd tertile	BF	148,084 (88.5)	19,301 (**11.5**)	148,084 (89.4)	17,576 (**10.6**)	148,084 (99.14)	1,289 (**0.86**)	148,084 (99.71)	436 (**0.29**)
	Non-BF	45,674 (89.0)	5,637 (**11.0**)	45,674 (90.1)	4,999 (**9.9**)	45,674 (99.02)	452 (**0.98**)	45,674 (99.59)	186 (**0.41**)
	Total	193,758 (88.6)	24,938 (11.4)	193,758 (89.6)	22,575 (10.4)	193,758 (99.11)	1,741 (0.89)	193,758 (99.68)	622 (0.32)

Table S3 of the Additional file 1 demonstrates the distribution of characteristics between beneficiaries and non-beneficiaries among preterm birth outcomes before the kernel procedure; they were similar across the four outcomes, but different when comparing beneficiaries and non-beneficiaries. With regards to sociodemographic and gestational characteristics, approximately 80% of beneficiaries had been enrolled in the cohort for at least five years; 37% had a low level of education; 76% were black or of mixed-race, beneficiaries include 4 times more indigenous people than non-beneficiaries; 67% were of childbearing age (20–35 years); 77% received adequate prenatal care; and 53% had vaginal deliveries (1.3 times higher than non-beneficiaries). Although 46% of the beneficiaries live in the northeast and 66% in urban areas, the remaining beneficiaries’ household characteristics were worse than those of non-beneficiaries.

The differences between beneficiaries and non-beneficiaries were minimized after weighting (Table 3; Table S4 in Additional file 1), and there was sufficient overlap in the PS of beneficiaries and non-beneficiaries according to overall (Figure S1 in Additional file 1) and subgroup analyses (Figures S2 and S3 in Additional file 1).

**Table 3:** Summarized statistics of the propensity score variables of beneficiaries (BF, exposed) and non-beneficiaries (non-BF, unexposed) before and after the kernel matching procedure. Brazil, 2004–2015

Propensity score variables	Relative frequency and non-weighted proportion			Relative frequency and weighted proportion		
	BF (*N* = 679,365)	Non-BF (*N* = 351,688)	Diff^1^	BF (*N* = 573,194)	Non-BF (*N* = 281,542)	Diff^2^
***Sociodemographic characteristics***						
**Cohort time (years)**						
≥ 5	79.8	65.6	14.2	82.0	82.5	-0.5
< 5	20.2	34.4	-14.2	18.0	17.5	0.5
**Maternal education (years)**						
0–7	37.2	21.9	15.3	37.3	37.4	-0.1
8–11	59.5	67.0	-7.5	59.5	59.5	0.0
≥ 12	3.3	11.1	-7.8	3.2	3.1	0.1
**Maternal race/skin color**						
White	23.2	37.5	-14.3	23.5	22.9	0.6
Black or mixed-race	75.6	62.1	13.5	75.5	76.2	-0.7
Indigenous	1.2	0.3	0.9	1.0	0.9	0.1
**Marital status**						
Marriage/civil partnership	55.2	59.5	-4.3	55.0	54.4	0.6
Single/divorced/widow	44.8	40.5	4.3	45.0	45.6	-0.6
***Household characteristics***						
**Geographical region**						
South	6.7	14.0	-7.3	6.9	6.9	0.0
North	17.0	11.4	5.6	16.9	16.9	0.0
Northeast	45.6	27.4	18.2	45.2	45.0	0.2
Southeast	23.9	36.1	-12.2	24.1	24.2	-0.1
Midwest	6.8	11.1	-4.3	6.9	7.0	-0.1
**Household location**						
Urban	66.4	81.4	-15.0	67.0	67.9	-0.9
Rural	33.6	18.6	15.0	33.0	32.1	0.9
**Household conditions**						0.0
All favorable conditions	24.1	40.2	-16.1	24.2	24.3	-0.1
1 unfavorable condition	22.2	27.2	-5.0	22.5	22.9	-0.4
2 unfavorable conditions	16.3	14.5	1.8	16.5	16.3	0.2
3 unfavorable conditions	15.8	10.0	5.8	15.7	15.3	0.4
4–5 unfavorable conditions	21.6	8.1	13.5	21.3	21.2	0.1
**Overcrowding**						0.0
≤ 2 people per room	87.8	95.0	-7.2	87.7	87.8	-0.1
> 2 people per room	12.2	5.0	7.2	12.3	12.2	0.1

^1^ Difference in the proportion of each category between Bolsa Familia beneficiaries and non-beneficiaries **before** kernel matching

^2^ Difference in the proportion of each category between Bolsa Familia beneficiaries and non-beneficiaries **after** kernel matching

The association between BF and PTB using weighted logistic regression analysis is presented in Table 4. In the adjusted model, there was no association between BF and overall, moderate-to-late, and severe PTB among Brazil and all subgroups analyzed, but it was associated with extreme PTB (Brazil weighted odds ratio [OR]: 0.69; 95% CI: 0.63–0.76). These findings were consistent across the adequacy of prenatal care levels and DMI, but the magnitude varied.

Although there was an association between BF and extreme PTB in both those receiving adequate and inadequate prenatal care, there seemed to be a potential modification of the association based on the adequacy of prenatal attendance. BF mothers receiving adequate prenatal care experienced a 34% reduction in extreme PTB (weighted OR: 0.66; 95% CI: 0.59–0.74), compared to 25% among those receiving inadequate prenatal care (weighted OR: 0.75; 95% CI: 0.62–0.90).

The association between BF and lower occurrence of extreme PTB varied according to the DMI level, ranging from 25% (weighted OR: 0.75; 95% CI: 0.65–0.86) in the worst tertile to 44% (weighted OR: 0.56; 95% CI: 0.43–0.74) in the best tertile (Table 4).

In the interaction analysis (Tables S6 and S7 in Additional file 1), considering each PTB outcome, the results of the likelihood ratio test indicated our interaction terms to be not statistically significant for BF status x prenatal care adequacy and BF status x DMI tertile.

As illustrated in Figure S4 (Additional file 1), the predictive margins for probability of preterm births have not indicated noticeable heterogeneity across subgroups. The likelihood ratio test comparing the models with versus without the interaction terms was not significant in all subgroup models (Tables S6 and S7 in Additional file 1).

Robustness analysis with IPTW yielded similar results to the kernel-weighted analysis, adding an association between BF and lower occurrence of severe PTB among mothers living in municipalities where the programme is best managed (weighted OR: 0.83; 95% CI: 0.72–0.95 - Table 4). The case-control analysis also found no association between BF and severe PTB (adjusted OR: 0.97; 95% CI: 0.89–1.06), being the association with extreme PTB attenuated but still strong (adjusted OR: 0.72; 95% CI: 0.63–0.83) when compared to the kernel-weighted analysis (Table S5 in Additional file 1).

**Table 4:** Coefficients of adjusted kernel-weighted logistic regressions of BF Programme benefit throughout pregnancy for preterm birth outcomes in Brazil, between 2012–2015, in accordance with adequacy of prenatal care and municipal BF decentralized management index (DMI) subgroups

Kernel matching	Weighted OR (95% CI) ^1^					
Outcome	Brazil	Adequate prenatal care	Inadequate prenatal care	1st tertile (worst) of the DMI	2nd tertile of the DMI	3rd tertile (best) of the DMI
All preterm birth (PTB)	0.98 (0.96-1.00)	0.99 (0.96–1.01)	0.98 (0.94–1.03)	0.99 (0.96–1.01)	0.98 (0.95–1.02)	0.96 (0.92–1.02)
N^2^	853,218	659,609	167,254	366,474	308,561	177,980
Moderate-to-late PTB	1.00 (0.98–1.02)	1.00 (0.98–1.03)	1.00 (0.95–1.05)	1.00 (0.97–1.03)	1.00 (0.96–1.03)	0.98 (0.93–1.04)
N^2^	842,977	652,607	164,666	361,857	304,899	176,023
Severe PTB	0.94 (0.88-1.00)	0.95 (0.88–1.02)	0.91 (0.80–1.04)	0.93 (0.85–1.02)	0.99 (0.89–1.11)	0.93 (0.78–1.10)
N^2^	765,481	593,147	148,943	319,459	267,859	154,602
Extreme PTB	**0.69 (0.63–0.76)**	**0.66 (0.59–0.74)**	**0.75 (0.62–0.90)**	**0.75 (0.65–0.86)**	**0.68 (0.58–0.79)**	**0.56 (0.43–0.74)**
N^2^	761,150	590,135	147,785	317,608	266,429	153,707
**IPTW**	**Brazil**	**Adequate prenatal care**	**Inadequate prenatal care**	**1st tertile (worst) of the DMI**	**2nd tertile of the DMI**	**3rd tertile (best) of the DMI**
All preterm birth (PTB)	1.01 (0.99–1.02)	1.00 (0.98–1.02)	1.00 (0.97–1.05)	1.00 (0.98–1.03)	1.01 (0.98–1.04)	0.98 (0.93–1.02)
N^2^	1,029,107	793,848	202,542	437,874	373,073	218,139
Moderate-to-late PTB	1.02 (1.00-1.04)	1.02 (1.00-1.04)	1.02 (0.98–1.06)	1.02 (0.99–1.04)	1.02 (0.99–1.06)	1.01 (0.96–1.05)
N^2^	1,016,948	785,533	199,476	432,426	368,720	215,781
Severe PTB	0.96 (0.90–1.02)	0.94 (0.88–1.01)	0.95 (0.85–1.06)	0.97 (0.89–1.05)	1.03 (0.93–1.14)	**0.83 (0.72–0.95)**
N^2^	923,367	713,886	180,415	393,448	334,892	195,008
Extreme PTB	**0.69 (0.63–0.75)**	**0.65 (0.59–0.72)**	**0.75 (0.63–0.89)**	**0.71 (0.63–0.81)**	**0.69 (0.60–0.80)**	**0.64 (0.52–0.80)**
N^2^	918,218	710,329	179,019	391,180	333,127	193,892

All the analytical steps (propensity score estimation, kernel matching, and weighted logistic regression) were conducted separately within each adequacy of prenatal care and DMI levels, respectively. All the outcome’s analyses were conducted separately, within each of these levels, and adjusted for maternal age (years) and type of delivery

^1^ Model adjusted for maternal age (years) and type of delivery

^2^ Sample size following kernel matching and IPTW, respectively

## Discussion

Drawing from a large, longitudinal populational-based cohort study that utilized national Brazilian linked data from health and social administrative databases and employed appropriately rigorous methods, our study enabled the evaluation of the association between the Brazilian conditional cash transfer and the occurrence and severity of PTB outcomes. Our findings suggest for the first time that receiving BF during pregnancy is significantly associated with a lower probability of extreme PTBs, but not with less severe PTB forms. Such an association was also observed for those live births to mothers receiving adequate prenatal care and living in municipalities with a better-managed programme.

The lack of effect of the program on overall, moderate, and severe PTB may be attributed to the potential prevention of these outcomes through a national expansion of prenatal care, regardless of the program’s conditionality. The success of prenatal care in reducing these differences implies that the BF programme does not have additional protective impacts on these outcomes above and beyond existing, national prenatal care efforts. The reinforcement of prenatal care coverage in Brazil resulted from government initiatives such as the Prenatal Care and Birth Humanization Programme established in 2000 [63], and the Rede Cegonha Program launched in 2011 [68]. These programs aim to enhance access and the quality of care, particularly in impoverished regions, with a focus on reducing neonatal, infant, and maternal mortality. Following the implementation of Rede Cegonha, there was a noticeable increase in prenatal consultations and the diagnosis of clinical complications, indicating improved care quality and more effective risk screening. This includes early identification and referral for high-risk pregnant women within the public health network. Additionally, in 2013, the Brazilian Ministry of Health released a technical manual to support healthcare teams involved in Rede Cegonha, focusing on low-risk prenatal care [69]. Despite the near-universal expansion of prenatal care access in Brazil through these government programs, the quality of care still exhibits inadequacies and inequities [70]. Table 2 shows that the percentages of adequate prenatal care for BF and non-BF recipients are very similar, suggesting that PTB outcomes preventable by prenatal care had already been addressed by primary care coverage and Rede Cegonha. Consequently, there was no significant difference in these outcomes comparing BF and non-BF recipients. However, extremely PTB remained a “less preventable” outcome. This implies that this particular outcome cannot be randomly mitigated; instead, it requires the provision of prenatal care, facilitating timely referrals for high-risk pregnancies.

To the best of our knowledge, our results add to the scant evidence base on the influences of social policies on PTBs concentrated on low-income populations in high-income countries. Three Canadian studies which evaluated an unconditional cash transfer programme - the Healthy Baby Prenatal Benefit - in low-income pregnant women [26] and indigenous women [40, 41] found a lower likelihood of a PTB [adjusted relative risks (aRR): 0.76; 95% CI: 0.69–0.84 [26], aRR: 0.78; 95% CI: 0.65–0.94 [40], and aRR: 0.77; 95% CI: 0.68–0.88 [41], respectively]. In the US, the participation of expectant mothers in the Special Supplemental Nutrition Program for Women, Infants, and Children (WIC) decreased the likelihood of a PTB (adjusted OR: 0.87; 95% CI: 0.86–0.87) [42]. Nevertheless, the EITC, recognized as the largest and most effective antipoverty program for families in the US, has shown a positive impact across racial and ethnic subgroups, leading to a decreased incidence of low birth weight and improvements in both mean birth weight and gestational duration [38, 39]. These studies did not address conditional programs, evaluate conditionalities, or explore the severity levels of PTBs. The differential which favours these studies in relation to our research was the long follow-up period and availability of an extensive array of maternal pre-and postnatal biological and social risk factors rarely available in administrative data.

The only study that found evidence on CCT on PTB in an LMIC was recently published in Brazil [43]. It indicated a higher probability of grandmothers receiving BF support during their childhood (measured as the prevalence of BF in the decade before child’s birth) being associated with a lower probability of low and very low birth weight. However, no trend was noted for PTB. Despite this, in households where the mother received BF, children were slightly less likely to be born preterm (OR 0.98, 95% CI; 0.97–0.99) - although the coefficient and the 95% confidence interval are close to one - or extreme preterm (OR 0.93, 95% CI; 0.88–0.97). While we did not find a significant association with the overall PTB group, our study revealed a notably stronger association with extreme PTB. This is possibly a consequence of the difference in methodological strategy, due to the definition of PTB outcomes and study population. It is crucial to note that they included all live births between 2011 and 2015 from both primiparous and multiparous mothers, thereby equalizing the program’s effect across different birth orders. Considering the pronounced associations between primiparity [48, 49], a higher number of pregnancies [49], multiple births [54], and congenital malformations [53] with PTB, these factors might have diluted the observed effect of the programme. It remains challenging to determine the extent to which this association can be solely attributed to the program or may be confounded by other factors. On the other hand, a longer exposure time to the program can positively influence better birth outcomes [71]. Consequently, it is not recommended to disregard the order of births by mixing, for instance, the first births after the mother’s enrollment and those born subsequently from the same mother throughout the follow-up.

The BF CCT might affect PTB through different mechanisms (Fig. 2). First, the income transfer increases household income, and improves nutrition, mitigating the effect of stressful life events. Second, by fulfilling the health conditionality, BF can improve access to and utilization of health services, strengthening beneficiary families’ contact with health services, especially primary health care; reducing barriers and improving prenatal visits, thereby amplifying the monitoring and treatment of comorbidities; facilitating timely referrals for high-risk pregnancies; and ensuring the provision of adequate assistance during childbirth. Third, the synergistic effect of these results may decrease the occurrence of PTBs [72]. Furthermore, the education and health-related conditionalities of BF encompass the requirement that all children must attend a minimum of 85% of school days, children aged 0–7 years and pregnant and lactating women must attend routine medical check-ups to monitor nutritional status and adhere to the national vaccination schedule. These conditionalities are based on the idea that making benefits conditional upon positive behaviours can further increase the chance of families breaking out the intergenerational cycle of poverty and inequalities through increased education, or improved health. For instance, increasing school attendance, and consequently improving educational levels, can also lead to improving the quality of social networks, i.e. reducing opportunities for certain types of crime and risky behaviour. The effect of increased use of health care has been suggested to extend beyond the individuals directly targeted by the programme [73].

Even though it is a conditionality, the effect of BF on PTB outcomes may vary according to prenatal care levels due to the non-immediate verification of compliance. While it is plausible that families adhering to BF conditionalities are more likely to comply with health care appointments compared to those not receiving BF, we do not expect this relationship to substantively confound the observed associations. Families are eligible for the BF benefit based on their socioeconomic need, and they would only lose the benefit after a minimum of two years of non-compliance with the conditions.

A recently published study on the effect of BF on birth weight-related outcomes [32] explored its association across subgroups based on attendance at prenatal appointments. The observed associations - increased birth weight and decreased odds of low birth weight among BF mothers who attended fewer prenatal appointments - suggested that beneficiaries and non-beneficiaries may share more similarities in characteristics not observed in subgroups of mothers at higher risk. These findings align with a recent review on CCTs and child health in LMICs, revealing considerable heterogeneity among subgroups based on socioeconomic status indicators [29]. 

As previously mentioned, given our primary findings and the substantial sample size, we chose to investigate the association between receiving BF during pregnancy and PTBs outcomes across subgroups based on prenatal care adequacy and municipal DMI, despite the lack of statistical significance in the interaction analysis. The analyzed prenatal care subgroups exhibited a trend consistent with our primary findings, suggesting that undergoing prenatal care, even if inadequate, has the potential to decrease the occurrence of extreme PTB. This reduction was even more pronounced in mothers who underwent adequate prenatal care. As extreme PTB is an outcome commonly associated with high-risk pregnancies, requiring timely healthcare assistance for early identification and referral within the health network, it is implied that BF recipients who adhere to healthcare appointments (serving as a marker for program participation, given the conditionality of BF) may experience a lower occurrence of extreme PTB compared to non-recipients, thereby enhancing programme management. In addition, we found evidence that being a BF recipient during pregnancy is associated with a lower risk of extreme PTBs across tertiles of municipal DMI - a broader contextual indicator that measures the quality of BF and CadÚnico administration. Consistent with our hypothesis, this association was stronger for beneficiaries living in municipalities where the programme has been best administered. In these cities, beneficiary children had lower odds of being born extremely preterm than their non-beneficiary counterparts. This heightened effect may be attributable to better-managed municipalities demonstrating greater adherence to program conditions, leading to increased inclusion of families in extreme poverty. Consequently, these municipalities provide enhanced access to, and superior quality of, primary health care. Within such well-organized health services with better resource allocation, more effective care ensues, ensuring that high-risk pregnancies receive appropriate and timely attention. This observation is particularly noteworthy within the context of the 100 M Brazilian Cohort, representing the most economically disadvantaged half of the nation. It underscores the pivotal significance not only of the presence but also the quality of poverty-alleviating policies when targeting health promotion among vulnerable populations. Our findings resonate with previous studies that underscored the dependence of cash transfers effect on implementation quality and management indicators [35, 74], reinforcing the validity and importance of our results.

### Strengths and limitations

Our study has notable strengths. First, we used propensity score-based approaches to evaluate the effect of BF on the first singleton birth following enrollment on CadÚnico. The study was based on a previously defined and published research protocol [46], providing transparency in conducting data analysis and comparability of the results. Second, we emphasize that comprehending this standardized categorization of PTB facilitates cross-population and research comparisons, thereby contributing to the formulation of health policy guidelines. Moreover, it plays a crucial role in guiding patient care strategies to prevent preterm-related morbidity and mortality [75]. Third, although our database reflects the poorest families in Brazil eligible for social programs, there were noteworthy distinctions between both eligible BF and non-BF groups, especially in terms of sociodemographic characteristics. These differences suggest that the benefits were targeted towards the most vulnerable. Therefore, we applied a robust analytical approach using kernel-based PS weighting and IPTW, to address observed confounding factors. Beneficiary and non-beneficiary groups were well-balanced in terms of covariate distributions, confirming our key findings through two subgroup analyses. Fourth, the linkage was performed with a robust and accurate algorithm, developed in-house by a specialized team [56–58]. 

The limitations should be considered. While the 100 M Cohort serves as a robust repository of sociodemographic information, it exclusively includes data from the low- and extremely low-income half of the population. Consequently, the results may not be fully representative of the entire population. Moreover, the approach employed could potentially underestimate the impact, as it may not account for women or families facing even greater disadvantaged who have not applied for the program. The external validity of the study was affected by the choice of the population since we only consider one child per woman. BF is used as a binary variable, and nuances related to the value received and levels of poverty were not investigated. Since we evaluated only live births, the highest rates of extreme PTB among non-beneficiaries may be due to the beneficiary had more stillbirths (that are not considered in the analyses). It is plausible that outcomes associated with stillbirths and spontaneous abortions are attenuated when analysing the association of BF with PTB in more homogeneous subgroups. As this is a study with secondary data, important unmeasured factors could not be included, such as family income, comorbidities, and complications during pregnancy (i.e., infections, placental abruption, pre-eclampsia, smoking, alcohol, and drug use by the mother), maternal nutritional status, or whether the delivery was spontaneous or by medical indication. We have attempted to minimize this by using different analytical approaches and performing subgroups and sensitivity analyses to strengthen the evidence produced.

## Conclusions

We provide new evidence that CCT programmes such as the BF, which supports vulnerable pregnant women, has been associated with lower rates of extreme preterm births, including those receiving adequate prenatal care and living where the programme is properly managed. This was the first assessment of the association between BF pregnant women and the highest severity of preterm births, based on large volumes of individual-level data and whereas only the first live child of a multiparous mother was included during the follow-up period. Public health and social inclusion policies are not only essential tools to improve the well-being of poor families, but also essential components to achieve the Sustainable Development Goals (SDGs), directly related to the eradication of poverty (Goal 1) and the reduction of inequality between and within countries (Goal 10), and indirectly to health and wellbeing (Goal 3) and access to justice, public security, and the promotion of a peaceful society (Goal 16). We also highlight the need to assess the association between participation in the BF and the occurrence of stillbirths, abortions, and infant survival.

### Electronic supplementary material

Below is the link to the electronic supplementary material.

Supplementary Material 1

Supplementary Material 2

## Data Availability

All data supporting the findings presented here used linked data from the 100 Million Brazilian Cohort and SINASC, coordinated and hosted by the Center for Data and Knowledge Integration for Health (CIDACS). In accordance with the policy of CIDACS and the Ministry of Health and Ministry of Citizenship (database providers), restrictions on the availability of this data apply. Currently, only national and international researchers who collaborate with CIDACS and authorized staff from government agencies can access de-identified or anonymized linked data. Any person who wishes to receive authorization must: (i) be affiliated with CIDACS or be accepted as a collaborator; (ii) present a detailed research project together with approval by an appropriate Brazilian institutional research ethical committee; (iii) provide a clear data plan restricted to the objectives of the proposed study and a summary of the analyses plan intended to guide the linkage and or data extraction of the relevant set of records and variables; (iv) sign terms of responsibility regarding the access and use of data; and (v) perform the analyses of datasets provided using the CIDACS data environment, a safe and secure infrastructure that provides remote access to de-identified or anonymized datasets and analysis tools. For more information, please visit the CIDACS website [https://cidacs.bahia.fiocruz.br/] or contact us via email [cidacs@fiocruz.br].

## Abbreviations

PTB: Preterm birth
CCT: Conditional Cash Transfer
BF: Bolsa Familia (Programme)
LMICs: Low and–middle–income countries
JSY: India’s Janani Suraksha Yojana
EITC: Earned Income Tax Credit
US: United States
100M Cohort: 100 Million Brazilian Cohort
CadÚnico: Unified Registry for Social Programs
SINASC: Live Birth Information System
CIDACS: Center for Data and Knowledge Integration for Health
CIDACS-RL: CIDACS Record Linkage
PHPN: Prenatal Care and Birth Humanization Programme
PS: Propensity Score
DMI: (municipal) Decentralized Management Index of the Bolsa Familia Programme and Unified Registry for Social Programs
IPTW: Inverse Probability of Treatment Weighting
SDG: Sustainable Development Goals
aRR: Adjusted relative risks
WIC: Special Supplemental Nutrition Program for Women, Infants, and Children
