# Supplementary material for: Evaluating the relationship between conditional cash transfer programme on preterm births: a retrospective longitudinal study using the 100 million Brazilian cohort
Source: BMC Public Health. 2024 Mar 5;24:713. doi: 10.1186/s12889-024-18152-2 (PMC10916064; doi:10.1186/s12889-024-18152-2)
Supplement: Supplementary file 2 — Supplementary Material 2 [file 12889_2024_18152_MOESM2_ESM.docx]

**Additional file 1**

*Evaluating the relationship between conditional cash transfer programme on preterm births: a retrospective longitudinal study using the 100 Million Brazilian Cohort*

**Definition of the study sample**

While information regarding children born prior to cohort enrollment is unavailable, we gathered data on the number of living and deceased children from the live birth certificate when the mother registered in the cohort. This allowed the classification of mothers as either primiparous or multiparous, and we obtained the birth order of the children from the original dataset. Consequently, we can assert that between 2004-2011, these mothers did not experience additional pregnancies. Then, we retained only the first singleton child born after the multiparous mother's enrollment but born from 2012 onward. In the Table S1 we provide information about the parity variable before excluding other children from primiparous or multiparous mothers with two or more children in the cohort.

Given the strong association between primiparity and preterm birth,^1,2^ we would be unable to ascertain how much of the observed effect on preterm birth could be attributed solely to the program or might be confounded by primiparity. On the other hand, around 24% of mothers who already had at least one child before cohort enrollment had only one child between 2004-2015 (born after 2012) (Table S1). Even with the possibility of creating the interpartal interval variable (and also estimating the intergestational interval) and recurrent prematurity, we deemed it unfair to overlook the order of births by mixing, in the analyses, the first births after the mother's enrollment and those born subsequently from the same mother throughout the follow-up. This is because a longer exposure time in the program is associated with better birth outcomes, and failing to distinguish between these groups would impede us from accurately measuring this difference. For instance, beneficiary mothers who already have a child during the cohort have prior experience related to prenatal care (both in terms of access to health facilities, medical teams, and information acquired in previous pregnancies).

For these reasons, we decided to include only the first singleton births during the study period (2004-2015), but born from 2012 onward, to multiparous mothers (who had at least one child before cohort enrolment).

**Characteristics of Variable Benefits for Breastfeeding Mothers (BVN) and Pregnant Women (BVG)**

In November and December 2011, the variable benefit for breastfeeding mothers (intended for families with children up to six months old) and pregnant women (aged 14 to 44) were introduced, respectively. These benefits are provided in six and nine monthly installments, each equivalent to the current variable benefit. Families receive these benefits only if they have not exceeded the limit of five variable benefits, despite meeting the eligibility criteria. Upon approval, pregnant women are required to undergo mandatory examinations and consultations. For breastfeeding mothers, it is essential that children under six months have an updated vaccination schedule and maintain regular nutritional monitoring.^3^ Unfortunately, information regarding these benefits is unavailable in our database.

**Interaction test**

To justify our hypothesis that the association between BFP and PTB outcomes varies across subgroups, we performed a statistical test for interaction by including BFP*subgroup indicator terms in all our subgroup models. The general results, including the likelihood ratio (LR) test to evaluate the interaction term, can be found on Tables S6 and S7.

**Supplementary Tables and Figures**

**Table S1.** Distribution (N, %) of the parity variable before excluding children born to both primiparous and multiparous mothers with two or more births in the cohort follow-up but born from 2012 onward.

| **Parity (i)** | **N** | **%** |
| --- | --- | --- |
| 0=Primiparous (1st child born after cohort enrollment) | 1,485,664 | 32.14 |
| 1=Multiparous - only child 1 born during the cohort period | 1,031,053 | 22.31 |
| 2=Multiparous - child 2 or more born during the cohort period | 2,105,197 | 45.55 |
| **Total** | **4,621,914** | **100,00** |
| **Parity (ii)** |  |  |
| 0=Primiparous (1st child born after cohort enrollment) | 1,485,664 | 32.14 |
| 1=Multiparous - only child born in the cohort period (1st child = last) | 1,116,844 | 24.16 |
| 2=Multiparous - child 2 or more born during the cohort period | 2,019,406 | 43.69 |
| **Total** | **4,621,914** | **100,00** |

**Table S2.** Accuracy results using best threshold, and number and percentage of linked records of live births system (SINASC) by year, Brazil, 2004-2015.

| **Year** | **Threshold** | **Specificity (%)** | **Sensibility (%)** | **Number of records SINASC^1^** | **Linked records (SINASC-CadÚnico) by year^2^** | |
| --- | --- | --- | --- | --- | --- | --- |
|  |  |  |  |  | **N** | **%** |
| 2004 | 0,954 | 98,4 | 94,6 | 3.026.548 | 1.501.466 | 49,6 |
| 2005 | 0,947 | 97,4 | 96,1 | 3.035.096 | 1.571.316 | 51,8 |
| 2006 | 0,915 | 81,6 | 96,4 | 2.944.928 | 1.839.816 | 62,5 |
| 2007 | 0,954 | 90,3 | 96,1 | 2.891.328 | 1.519.251 | 52,5 |
| 2008 | 0,955 | 88,7 | 97,7 | 2.934.828 | 1.534.826 | 52,3 |
| 2009 | 0,950 | 87,4 | 98,2 | 2.881.581 | 1.526.931 | 53,0 |
| 2010 | 0,944 | 93,5 | 98,6 | 2.861.868 | 1.497.939 | 52,3 |
| 2011 | 0,955 | 86,6 | 97,6 | 2.913.160 | 1.710.423 | 58,7 |
| 2012 | 0,925 | 88,1 | 97,4 | 2.905.789 | 1.757.979 | 60,5 |
| 2013 | 0,952 | 88,5 | 98,6 | 2.904.027 | 1.919.632 | 66,1 |
| 2014 | 0,953 | 86,7 | 97,9 | 2.979.259 | 1.993.386 | 66,9 |
| 2015 | 0,955 | 88,3 | 98,4 | 3.017.668 | 1.859.496 | 61,6 |
| **2004-2015** |  |  |  | **35.296.080**** | **20.232.461**** | **57,3*** |

*Average for the period.

** Sum for the period.

^1^ Information obtained from the DATASUS website: http://tabnet.datasus.gov.br/cgi/tabcgi.exe?sinasc/cnv/nvuf.def

^2^ Linkage report between the POP100V2 and SINASC datasets.

**Table S3.** Sociodemographic, gestational, and household characteristics in relation to preterm birth outcomes and whether or not the mother received Bolsa Familia (BF) during pregnancy.

(In the excel file)

**Table S4.** Summarized statistics of the propensity score variables of beneficiaries (exposed) and non-beneficiaries (unexposed) before and after the kernel matching, according to preterm birth outcomes.

| **Propensity score variables** | **Outcome 1^a^** | | **Outcome 2^b^** | | **Outcome 3^c^** | | **Outcome 4^d^** | |
| --- | --- | --- | --- | --- | --- | --- | --- | --- |
|  | *Diff* ***before*** *Kernel^1^* | *Diff* ***after*** *Kernel^2^* | *Diff* ***before*** *Kernel^1^* | *Diff* ***after*** *Kernel^2^* | *Diff* ***before*** *Kernel^1^* | *Diff* ***after*** *Kernel^2^* | *Diff* ***before*** *Kernel^1^* | *Diff* ***after*** *Kernel^2^* |
| **Cohort time (years)** |  |  |  |  |  |  |  |  |
| ≥ 5 | 13.8 | -1.1 | 14.2 | -0.9 | 12.4 | -1.1 | 8.3 | -6.3 |
| <5 | -13.8 | 1.1 | -14.2 | 0.9 | -12.4 | 1.1 | -8.9 | 6.3 |
| **Maternal education (years)** |  |  |  |  |  |  |  |  |
| 0-7 | 16.4 | -1.3 | 16.4 | -1.2 | 15.5 | -1.7 | 13.9 | -1.9 |
| 8-11 | -8.8 | 1.3 | -8.8 | 1.2 | -7.0 | 2.1 | -7.1 | 2.2 |
| ≥ 12 | -7.6 | 0.0 | -7.6 | 0.0 | -8.5 | -0.4 | -6.8 | -0.2 |
| **Maternal race/skin color** |  |  |  |  |  |  |  |  |
| White | -14 | 1.2 | -14.1 | 1.5 | -14.4 | -1.0 | -12.4 | 0.1 |
| Black or mixed-race | 12.7 | -1.2 | 12.8 | -1.5 | 13.1 | 0.0 | 11.7 | 0.0 |
| Indigenous | 1.3 | 0.1 | 1.3 | 0.0 | 1.3 | 1.0 | 0.7 | 0.0 |
| **Marital status** |  |  |  |  |  |  |  |  |
| Marriage/civil partnership | -4.5 | 0.6 | -4.5 | 0.6 | -6.1 | -1.4 | -1.0 | 2.7 |
| Single/divorced/widow | 4.5 | -0.6 | 4.5 | -0.6 | 6.1 | 1.4 | 1.0 | -2.7 |
| **Geographical region** |  |  |  |  |  |  |  |  |
| South | -6.7 | 0.1 | -5.8 | 0.2 | -7.0 | -0.1 | -6.2 | 0.2 |
| North | 7.3 | 0.6 | 7.3 | 0.3 | 8.5 | 3.8 | 6.0 | 2.1 |
| Northeast | 17.1 | -0.4 | 17.2 | -0.3 | 15.1 | -2.0 | 17.3 | -1.7 |
| Southeast | -12.9 | -0.1 | -12.9 | 0.1 | -11.7 | -1.7 | -14.0 | -0.3 |
| Midwest | -4.7 | -0.3 | -4.8 | -0.4 | -4.8 | 0.1 | -3.1 | -0.2 |
| **Household location** |  |  |  |  |  |  |  |  |
| Urban | -15.2 | -1.0 | -15.3 | -0.8 | -14.7 | -1.1 | -13.1 | -1.7 |
| Rural | 15.2 | 1.0 | 15.3 | 1.4 | 14.7 | 1.1 | 13.1 | 1.7 |
| **Household conditions** |  |  |  |  |  |  |  |  |
| All favorable conditions | -17.0 | -0.1 | -17.1 | 0.0 | -16.6 | -0.8 | -15.6 | -0.5 |
| 1 unfavorable condition | -5.5 | -0.5 | -5.7 | -0.7 | -3.5 | 1.3 | -4.5 | -1.5 |
| 2 unfavorable conditions | 2.1 | 0.3 | 2.2 | 0.4 | 0.9 | -2.0 | 6.1 | 4.0 |
| 3 unfavorable conditions | 5.8 | 0.6 | 5.8 | 0.6 | 5.4 | -0.1 | 5.5 | 0.9 |
| 4-5 unfavorable conditions | 14.5 | -0.2 | 14.7 | -0.3 | 13.8 | 1.6 | 8.5 | -2.8 |
| **Overcrowding** |  |  |  |  |  |  |  |  |
| ≤2 people per room | -8.6 | -0.5 | -8.6 | -0.2 | -8.7 | -2.6 | -8.5 | -3.2 |
| >2 people per room | 8.6 | 0.5 | 8.6 | 0.2 | 8.7 | 2.6 | 8.5 | 3.1 |

^a^ 1= all preterm births (PTB), 0=at term newborns.

^b^ 1= moderate-to-late PTB, 0=at term newborns.

^c^ 1=severe PTB, 0=at term newborns.

^d^ 1=extreme PTB, 0=at term newborns.

^1^ Difference between beneficiaries and non-beneficiaries (BF1-BF0) **before** kernel matching.

^2^ Difference between beneficiaries and non-beneficiaries (BF1-BF0) **after** kernel matching


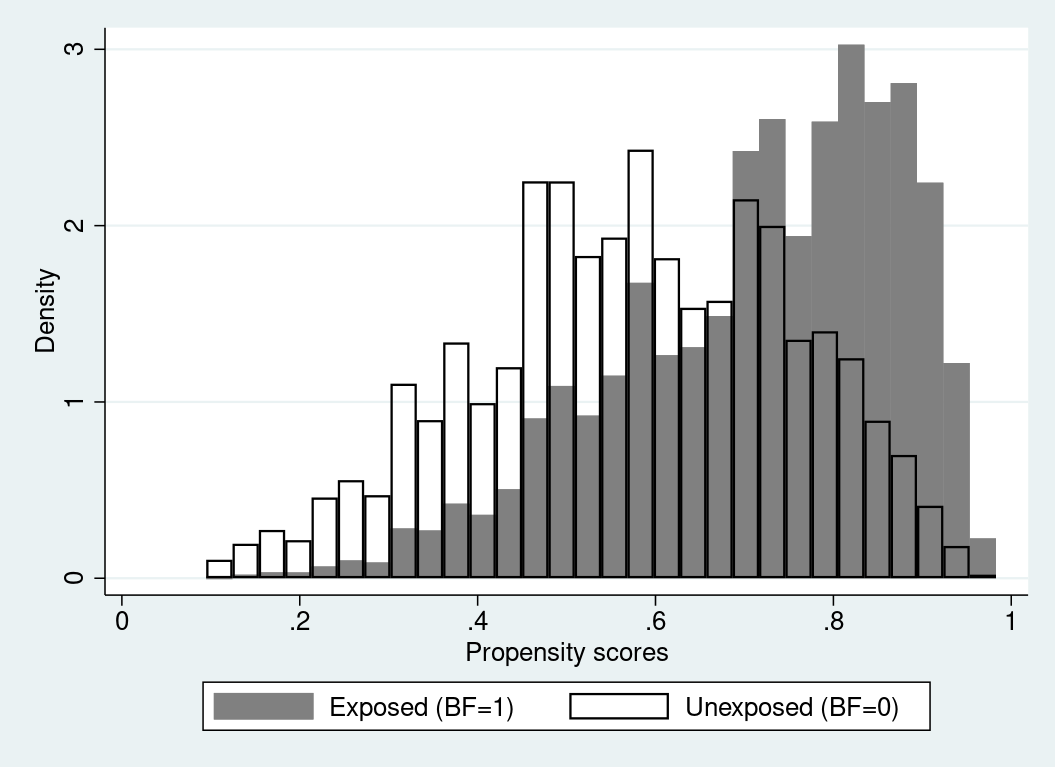


**Figure S1.** Common support area of the exposed over the unexposed group.


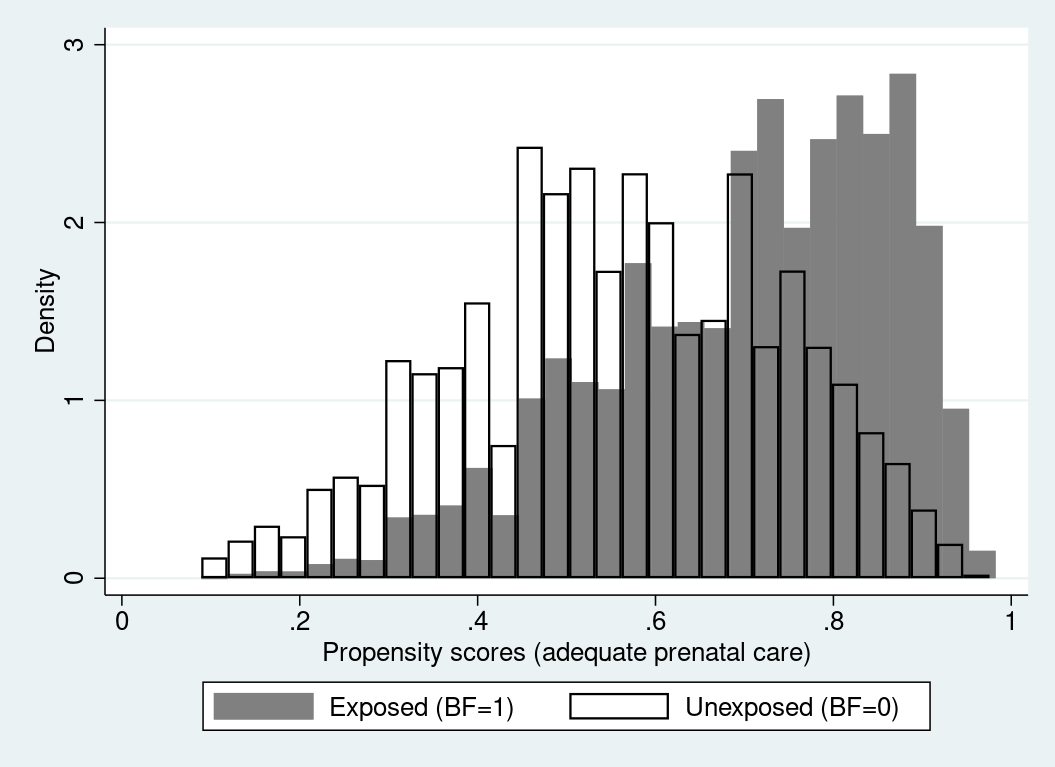


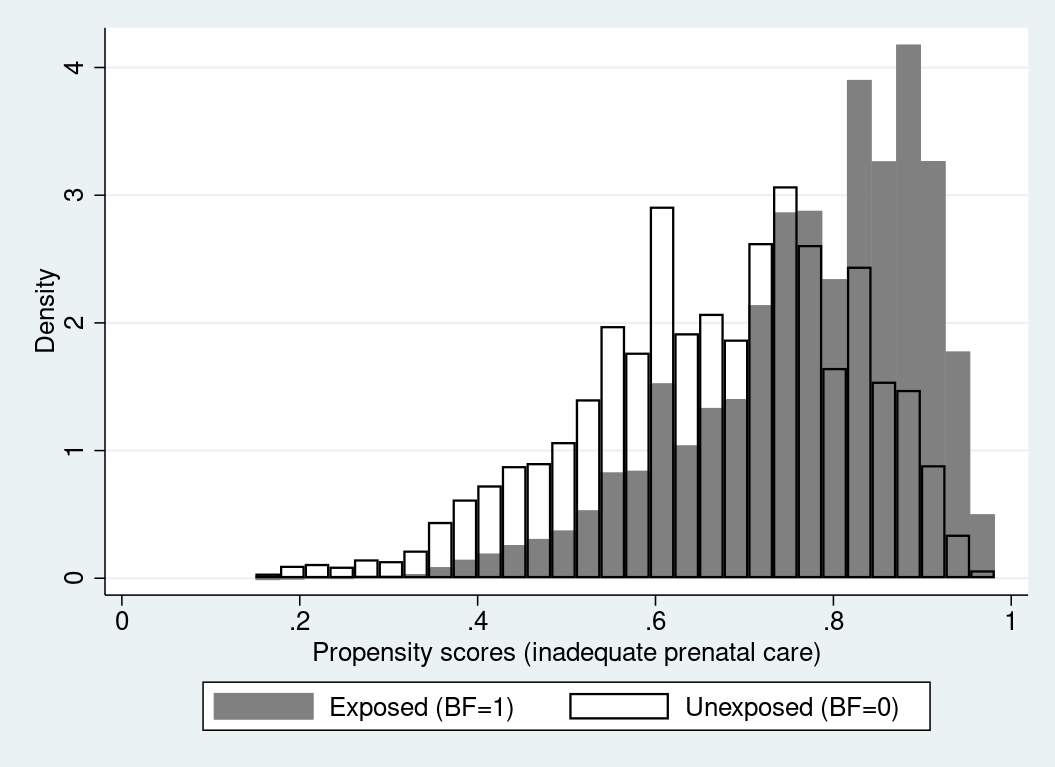
**Figure S2.** Common support area of the exposed over the unexposed group, according to adequacy of prenatal care subgroups.


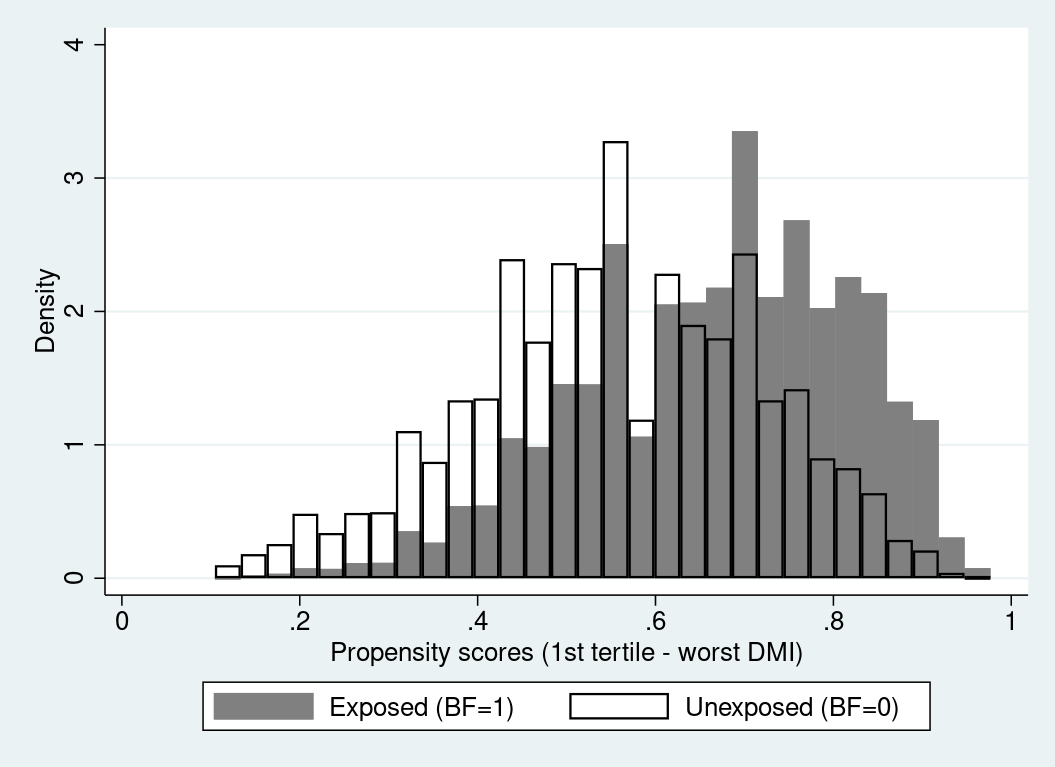

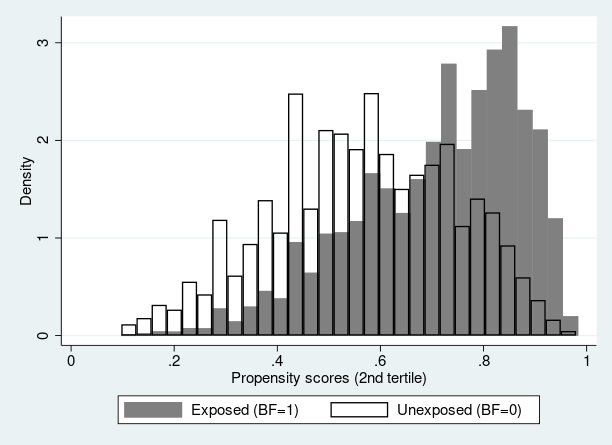

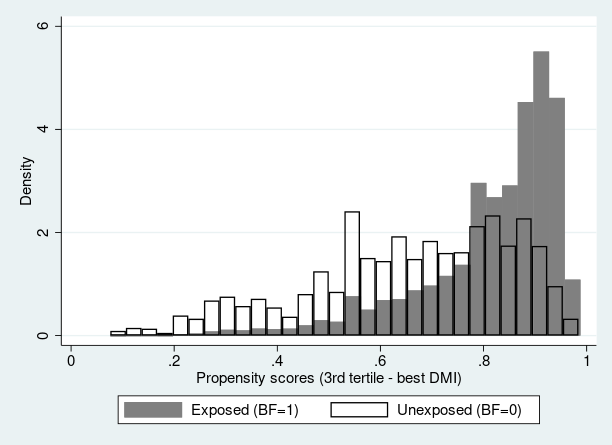


**Figure S3.** Common support area of the exposed over the unexposed group, according to municipal Bolsa Familia (BF) Programme Decentralized Management Index (DMI) subgroups.

**Table S5.** Association between Bolsa Familia (BF) Programme benefit during pregnancy and rare preterm birth outcomes.

| **Rare preterm birth outcome** | **OR (95% CI)^1^** |
| --- | --- |
| **Severe PTB** |  |
| No (37-42 weeks) | 1.00 |
| Yes (28-31 weeks) | 0.97 (0.89-1.06) |
| N^2^ | 9,978 |
| **Extreme PTB** |  |
| No (37-42 weeks) | 1.00 |
| Yes (<28 weeks) | **0.72 (0.63-0.83)** |
| N^2^ | 4,336 |

^1^Model adjusted for maternal age (years) and type of delivery.

^2^Sample size following case-control analysis.

**Table S6.** Regression results of interaction analysis. Coefficients on adjusted^1^ kernel weighted logistic regression for the association between Bolsa Familia (BF) Programme participation and preterm births (PTB) groups by prenatal care adequacy (prenatal) and BFxprenatal interaction term^2^.

| **Outcome** | **Prenatal care adequacy subgroup** | **Weighted Odds Ratio** | **Robust Standard error** | **p-value** |
| --- | --- | --- | --- | --- |
| PTB | Beneficiary status (BF=1) | 0.984 | 0.011 | 0.108 |
|  | BF x inadequate prenatal | 0.989 | 0.026 | 0.682 |
|  | LR test* = 0.58 (p-value = 0.445) | | | |
| Moderate-to-late PTB | Beneficiary status (BF=1) | 0.999 | 0.013 | 0.784 |
|  | BF x inadequate prenatal | 0.994 | 0.028 | 0.819 |
|  | LR test* = 0.19 (p-value = 0.665) | | | |
| Severe PTB | Beneficiary status (BF=1) | 0.946 | 0.037 | 0.052 |
|  | BF x inadequate prenatal | 0.959 | 0.075 | 0.596 |
|  | LR test* = 0.84 (p-value = 0.360) | | | |
| Extreme PTB | Beneficiary status (BF=1) | 0.665 | 0.038 | <0.001 |
|  | BF x inadequate prenatal | 1.116 | 0.128 | 0.338 |
|  | LR test* = 2.27 (p-value = 0.132) | | | |

^1^ The model was adjusted for maternal age (years) and type of delivery.

^2^ Model equation: BF + prenatal + BFxprenatal

Reference category = adequate prenatal care

*Model without interaction term x the model with interaction term.

**Table S7.** Regression results of interaction analysis. Coefficients on adjusted^1^ kernel weighted logistic regression for the association between Bolsa Familia (BF) Programme participation and preterm births (PTB) groups by tertiles of municipal Decentralized Management Index (DMI) and BFxDMI interaction term^2^.

| **Outcome** | **Municipal DMI subgroup** | **Weighted Odds Ratio** | **Robust Standard error** | **p-value** |
| --- | --- | --- | --- | --- |
| PTB | Beneficiary status (BF=1) | 0.980 | 0.014 | 0.059 |
|  | BF x DMI 2nd tertile | 1.006 | 0.022 | 0.999 |
|  | BF x DMI 3rd tertile (best) | 0.991 | 0.029 | 0.999 |
|  | LR test* = 0.84 (p-value = 0.658) | | | |
| Moderate-to-late PTB | Beneficiary status (BF=1) | 0.995 | 0.015 | 0.599 |
|  | BF x DMI 2nd tertile | 1.004 | 0.024 | 0.999 |
|  | BF x DMI 3rd tertile (best) | 0.990 | 0.031 | 0.999 |
|  | LR test* = 0.69 (p-value = 0.708) | | | |
| Severe PTB | Beneficiary status (BF=1) | 0.926 | 0.044 | 0.173 |
|  | BF x DMI 2nd tertile | 1.041 | 0.077 | 0.999 |
|  | BF x DMI 3rd tertile (best) | 1.040 | 0.098 | 0.999 |
|  | LR test* = 0.96 (p-value = 0.620) | | | |
| Extreme PTB | Beneficiary status (BF=1) | 0.744 | 0.051 | <0.001 |
|  | BF x DMI 2nd tertile | 0.926 | 0.094 | 0.894 |
|  | BF x DMI 3rd tertile (best) | 0.833 | 0.124 | 0.439 |
|  | LR test* = 4.72 (p-value = 0.095) | | | |

^1^ The model was adjusted for maternal age (years) and type of delivery.

^2^ Model equation: BF + DMI + BFxDMI

Reference category = 1^st^ tertile (worst)

*Model without interaction term x the model with interaction term.

**Figure S4.** Predictive margins for probability of preterm births (PTB) outcomes, with 95% of confidence intervals, by subgroup.

(b)


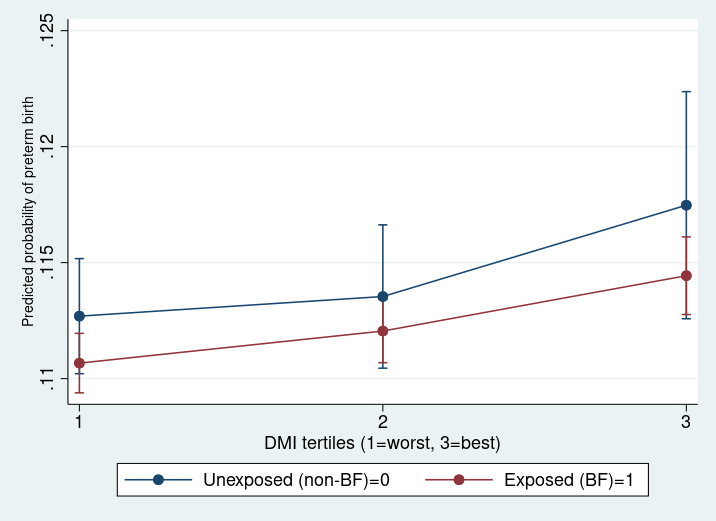


(a)


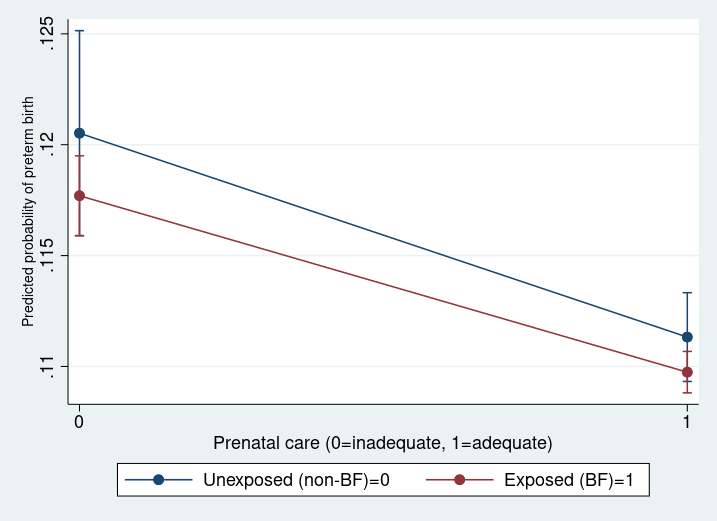

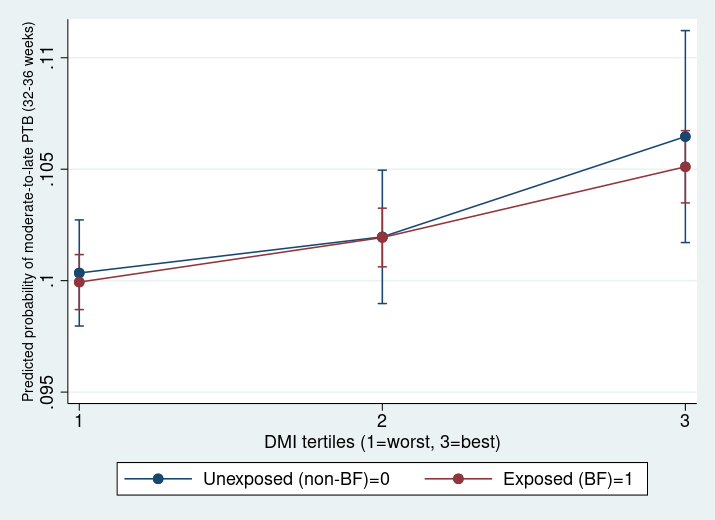


(d)


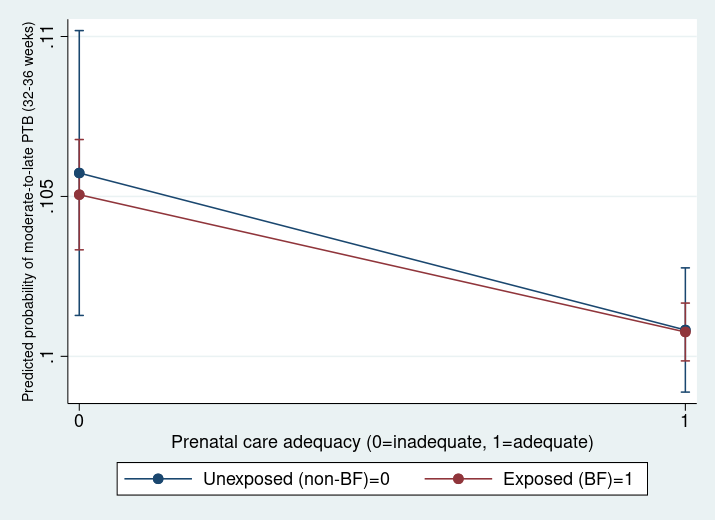


(c)


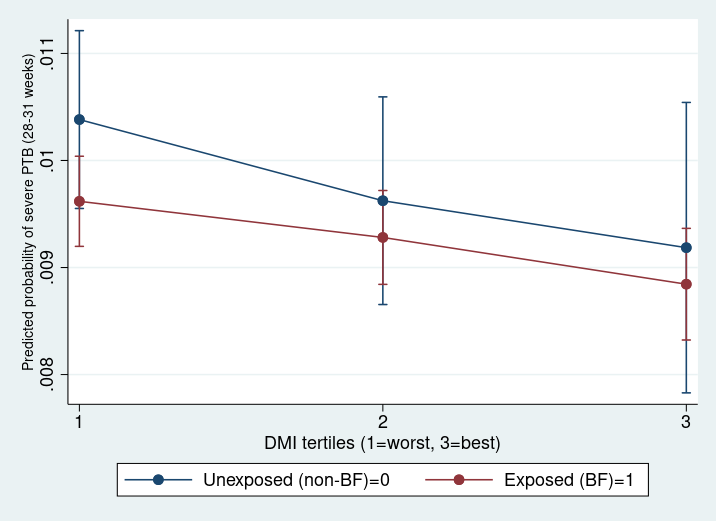


(f)

(e)


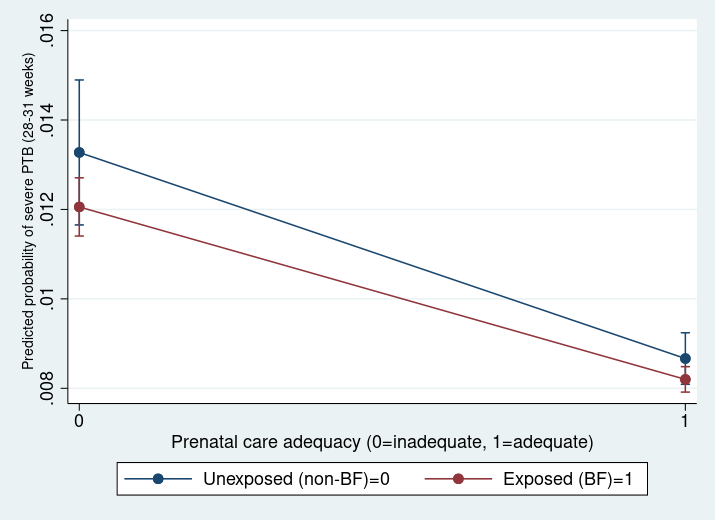


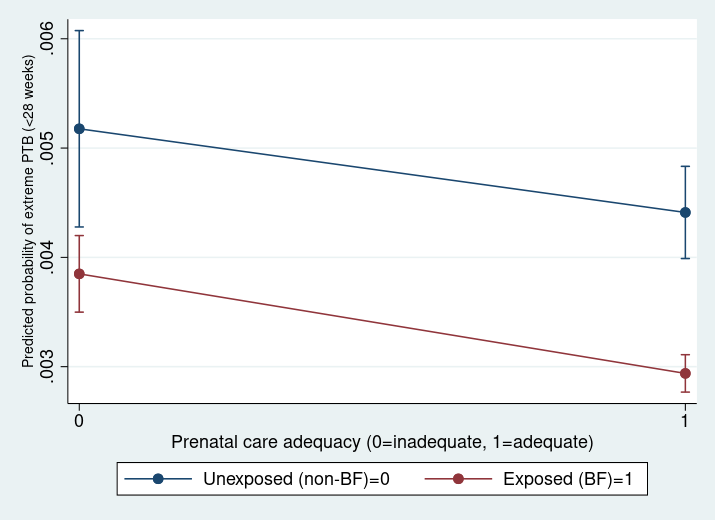


(g)


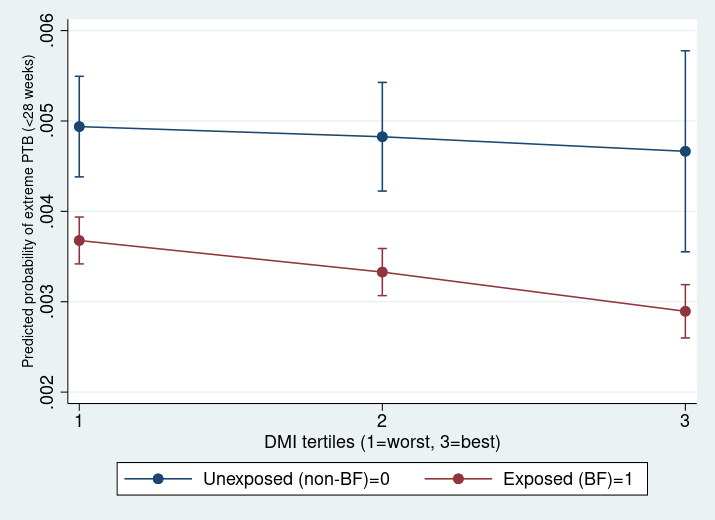


(h)

Beneficiary status: 0=unexposed (non-BF); 1= exposed (BF). (a) Predictive margins (95% CI) for probability of PTB (<37 weeks) by prenatal care adequacy. Model adjusted for maternal age (years) and type of delivery. (b) Predictive margins (95% CI) for probability of PTB (<37 weeks) by tertile of municipal Decentralized Management Index (DMI). Model adjusted for maternal age (years) and type of delivery. (c) Predictive margins (95% CI) for probability of moderate-to-late PTB (32-36 weeks) by prenatal care adequacy. Model adjusted for maternal age (years) and type of delivery. (d) Predictive margins (95% CI) for probability of moderate-to-late PTB (32-36 weeks) by tertile of municipal Decentralized Management Index (DMI). Model adjusted for maternal age (years) and type of delivery. (e) Predictive margins (95% CI) for probability of severe PTB (28-31 weeks) by prenatal care adequacy. Model adjusted for maternal age (years) and type of delivery. (f) Predictive margins (95% CI) for probability of severe PTB (28-31 weeks) by tertile of municipal Decentralized Management Index (DMI). Model adjusted for maternal age (years) and type of delivery. (g) Predictive margins (95% CI) for probability of extreme PTB (<28 weeks) by prenatal care adequacy. Model adjusted for maternal age (years) and type of delivery. (h) Predictive margins (95% CI) for probability of extreme PTB (<28 weeks) by tertile of municipal Decentralized Management Index (DMI). Model adjusted for maternal age (years) and type of delivery.

**References**

1. Ananth CV, Peltier MR, Getahun D, Kirby RS, Vintzileos AM. Primiparity: An ‘intermediate’ risk group for spontaneous and medically indicated preterm birth. J Matern Fetal Neonatal Med. 2007 Jan 1;20(8):605–11.

2. Koullali B, van Zijl MD, Kazemier BM, Oudijk MA, Mol BWJ, Pajkrt E, et al. The association between parity and spontaneous preterm birth: a population based study. BMC Pregnancy Childbirth. 2020 Apr 21;20(1):233.

3. Brasil. LEI N^o^ 14.601, DE 19 DE JUNHO DE 2023 - Institui o Programa Bolsa Família; altera a Lei n^o^ 8.742, de 7 de dezembro de 1993 (Lei Orgânica da Assistência Social), a Lei n^o^ 10.820, de 17 de dezembro de 2003, que dispõe sobre a autorização para desconto em folha de pagamento, e a Lei n^o^ 10.779, de 25 de novembro de 2003; e revoga dispositivos das Leis n^o^s 14.284, de 29 de dezembro de 2021, e 14.342, de 18 de maio de 2022, e a Medida Provisória n^o^ 1.155, de 1^o^ de janeiro de 2023. [Internet]. 2023 [cited 2023 Dec 14]. Available from: https://www.planalto.gov.br/ccivil_03/_ato2023-2026/2023/lei/l14601.htm
